# Nano eggshell-titanium-dioxide biocomposite in occluding opened dentine tubules

**DOI:** 10.6026/97320630018853

**Published:** 2022-10-31

**Authors:** Navjot Singh Mann, Ashu Jhamb, Nimrat Kaur, Navneet Mann

**Affiliations:** 1 Department of Conservative Dentistry and Endodontics, National Dental College, Derabassi, Mohali, Punjab, India

**Keywords:** Dentin, dentin sensitivity, dentin desensitizing agents, tooth remineralization

## Abstract

It is of interest to synthesize Nano eggshell-titanium-dioxide (EB@TiO2) biocomposite and to evaluate its effectiveness in occluding opened dentine tubules. EB@TiO2 was synthesized and characterized using X-ray diffraction (XRD), and Transmission Electron Microscope (TEM). Sixteen simulated bovine dentine discs were prepared and randomly assigned into four groups according to the following treatment (n = 4): Group 1: No treatment; Group 2: eggshell powder; Group 3: EB@TiO2; and Group 4: GIC mousse. These were then, agitated in a solution of 1g powder and 40mL water for 3hours. Thereafter, each dentine discs from the respective groups were post-treated for 5 min with 2wt% citric acid to test their acid resistant characteristics. Scanning Electron Microscope (SEM) was used to observe the effectiveness of occluded dentine pre-treatment and post-treatment. The cytotoxicity of the synthesized EB@TiO2 was tested using NIH 3T3 assay. ANOVA was used to evaluate the mean values of the occluded area ratio and the data of MTS assay. This was followed by a multi-comparison test with Bonferroni correction (α = .05). The XRD confirmed that EB@TiO2 was successfully modified through ball-milling. The TEM revealed the presence of both spherical and irregular particle shape powders. The SEM result showed that EB@TiO2 could effectively occlude open dentine tubules. Equally, the result demonstrated that EB@TiO2 exhibited the highest acid resistant stability post-treatment. NIH 3T3 assay identified that EB@TiO2 had little effect on the NIH 3T3 cell line even at the highest concentration of 100µg/ml. This study suggests that the application of EB@TiO2 effectively occluded dentine tubules and the occlusion showed a high acid resistant stability.

## Background:

Dentine hypersensitivity is a typical phenomenon in which individuals experience transient, intense discomfort when dentine tubules are introduced to the oral cavity [1]. DH is a serious public health problem since it affects greater than 43 percent of the adult population globally. As a result, DH, if left not treated, it will have a significant impact on dental patients' wellbeing [2]. As Schiff et al. note out, DH patients tend to change their behaviours by excluding some foods and beverages from their usual routine diets, and they become non-compliant with certain at-house care suggestions like as dental cleaning [3]. Yang et al. recommend utilising occlusion agents to physically plug open dentine tubules. The dental care industry has seen a surge of new occlusion materials for treating DH during the last few decades [4]. Potassium oxalates [5], sodium fluoride [6], barium salt [2], calcium phosphate containing casein phosphopeptide [7], calcium glycerophosphate [8], and calcium carbonate (mainly as abrasion agents [9] have received a lot of interest as occlusion materials. Even though the above occlusion substances have been observed to give some comfort to the patients, the dentine tubules occluded by some of these materials have been observed to be superficial with low infiltration depth-which might easily be re-exposed in an acidic environment [10]. As a result, the therapy results are short-lived, leading to DH recurrence [11]. Considering the few disadvantages, finding a desired biomaterial for DH becomes crucial in order to not only successfully occlude the exposed dentine tubules, but also to function effectively in an acidic environment. Eggshells are now being researched for their remineralization potential [12]. Now a day's lately, Haghgoo et al. [13] demonstrated that eggshell has a high concentration of bio available calcium, which promotes the remineralization of caries lesions. Cutler proposed combining nano sized titanium dioxide with dental abrasive agents for occluding open dentine tubules. Tooth Mousse is the topical tooth crèmes that helps to strengthen and rejuvenate the patient's teeth. Tooth Mousse is the first product for professional use to contain this complex of CPP-ACP (Recaldent™), which is the ideal delivery system for bio-available calcium and phosphate ions [14]. Considering the desired features of titanium dioxide (TiO2) and the remineralization potentials of eggshells (EB), a novel EB@TiO2 bio material will be critical in the treatment of DH. Despite its immense potential, there is no proof that eggshell-titanium dioxide bio material can be used to occlude dentine tubules. Therefore, it is of interest to synthesize Nano eggshell-titanium-dioxide (EB@TiO2) biocomposite and to evaluate its effectiveness in occluding opened dentine tubules.

## Methodology:

### Eggshell-Titanium dioxide composite (EB@TiO2) preparation:

The eggshell was modified with titanium dioxide in 2 phases. In the 1st phase, eggshells were ball-milled in a planetary ball mill (Retsch ® PM 100) at 400 rpm for 20 minutes by putting 30g of the eggshell in a 500ml stainless jar (inner diameter of the 100 mm) with 10 stainless steel ball of 10mm of diameter. Using a mechanical sieving shaker, the obtained powder was sieved to a particle size of 25m (Retsch AS 200, Germany). Lin et al. devised a technique for adjusting the mixing ratio of eggshell powder and titanium dioxide (16) In step 1, 5g of anatase titanium dioxide was added to 20g of fine eggshell powder (15m). To make an eggshell-titanium dioxide bio composite, the mixture was ball-milled for 200minute.

### Characterization of EB@TiO2:

Microscopic examination the matter size, shape, distribution of EB@TiO2 was studied using a Transmission Electron Microscope (TEM). Small amounts of EB@TiO2 were disseminated in 10mL ethanol and sonicated for 10 minutes at 10kV. Then, using a Leica microtome (South Africa), thin cross-sections of cryo-microtome specimens were produced and put on carbon copper grids. At 120 kV, an electron transmission microscope (TEM-Philips CM 120 model) was used for the analysis.

### Acidic challenge and specimen preparation:

16 freshly removed human enamel front teeth were procured, following that, the teeth were cleansed and disinfected in a Chloroxylenol solution at 10%. Dentin discs measuring 5mm length x 5mm breadth x 1mm width were made by segmenting below the adhesive interface with a reduced speed diamond saw while using liquid cooling system. Following that, produced dentin disc was wet ground for 60 seconds using silicon carbide polishing sheets (600-1,000 grits). The discs were put in a resin before replicating the sensitive tooth model (AMT composite, South Africa). A mounting platform was created using a silicone mould (Silicone rubber mould; Agar scientific). In a disposable plastic cup, a fast setting resin (F160: AMT composite) was mixed in a 1:1 ratio and poured into the mould. Approximately 2 minutes later, the silicone mold's imbedded resin was removed. The dentine tubules were then opened by immersing the specimens in a 1 wt. percent citric acid solution for 30 minutes. The specimens were divided into four groups (n = 4) at random, as follows: - Group 1: No treatment group, Group 2: The eggshell powder group, Groups 3: Treated with EB@TiO2 and Groups 4: Treated with, GIC Mousse. For 3 hours, the specimens were stirred in a beaker with 1g of synthesised EB@TiO2 and 40mL deionized water. Eggshell powder and GIC Mousse paste were treated in the same way. After that, the specimens were rinsed and blotted. A representative of each sample group was chosen as a proxy measure to determine the acid resistance qualities of the treated material. After that, they were subjected to a 2 percent citric acid solution (pH 2) for 5 minutes. After exposure, the specimens were cleaned with deionized water and blotted.

### Evaluation of the obstructed specimen using a scanning electron microscope:

The occluded dentine was evaluated using a scanning electron microscope (Field Emission-Carl Zeiss) operated at regulated air conditions at 20 kV. To minimise electrostatic charge build-up, the surface was covered with a thin, electric conductive gold coating prior to SEM inspection.

## Results:

### Characterization:

Figure 1(see PDF) shows the XRD pattern of the eggshell powdered titanium dioxide and it produced EB@TiO2. Figure 1A shows a thermodynamically stable calcite crystalline phase, which is identical to calcium carbonate, with a distinctive peak about 34.502. [17] The anatase phase is validated by the EB@TiO2 diffraction peak with values resting at 2 = 29.50, as confirmed by the International Centre for Diffraction Data (ICDD Ref: 98-009-6946). The form, intensity, and placement of the EB@TiO2 peaks corresponding to the anatase are consistent with Tao et al. [15] TiO2 deposition on the surface of CaCO3 is suggested. TEM was used to examine eggshell powder and EB@TiO2 form, shape, and particle size. The eggshell had an uneven particle form, as shown in the TEM picture in Figure 2A(see PDF). As seen in Figure 2B(see PDF), uneven particle morphologies lived side by side with spherical form particles. The irregular shaped particles were linked to the eggshell powder's calcite morphology, but the spherical shaped particles indicated the presence of TiO2.

### Cytotoxicity testing:

NIH 3T3 mouse fibroblast cell lines were used to evaluate the samples. There was no significant difference between the groups (p > 0.05). In all pair wise comparisons of the different concentrations, there were no significant differences (p > 0.05).

### Observation of the occluded dentine tubules:

Dentine tubules that have become blocked, Table1 show the 1-way ANOVA findings, including mean, standard deviation, and standard error. The mean ratio of occluded tubules values for the specimens treated with eggshell powder EB@TiO2, and GIC Mousse, for example, were significantly different (p.001). The specimens treated with EB@TiO2 had the largest mean occluded regions (82.14 13.32 m2), whereas the GIC Mousse group had the smallest open tubule coverage (26.00 7.64 m2) (Table 1 - see PDF). The area of tubules occluded by the EB@ TiO2 group was considerably larger than that of the eggshell powder group (p.001) and the GIC Mousse group (p.05), according to the findings of the post hoc comparison test (Table 2 - see PDF). In the occluded tubules, significant differences were identified in the groups treated with eggshell powder and GIC Mousse (p.05). Figure 4(see PDF) shows a SEM micrograph showing clogged dentine tubules before and after treatment in various sample groups. In group 1 (A1-B1), Following 30 minutes of agitation in 1 wt. percent citric acid, the dentine surfaces were free of smear layer and all dentine tubules were open. In group 2 (A2–B2), Dentine tubules were partly occluded, with some tubules visible to the naked eye. In group 3 (A3-B3), the particles of EB@TiO2 totally occluded the dentine tubules. In group 4 (A4-B4), After GIC Mousse treatment, the majority of the dentine tubules are still open (Table 2 - see PDF). Furthermore, the SEM picture (C2-C4) demonstrated variations in the treated dentine specimen after 2wt% citric acid treatment. In contrast to the photos of the specimens treated with eggshell powder (C2) and GIC Mousse (C4), the images of the specimen treated with EB@TiO2 (C3) show greater acidic challenge tolerance.

## Discussion:

Tooth erosion linked to increased intake of citric acid-containing soft drinks is increasingly considered as a public health problem, leading to DH, according to the research [15]. The effective occlusion of the dentine tubules is a viable method for DH therapy. The goal of this work was to make a ball-milled EB@TiO2 that could occlude open dentine tubules analyze its success in occluding tubules. In terms of the width of the dentine tubules, the radicular dentine morphology of human and bovine primary teeth and roots are very comparable [16-19]. The stated hypothesis was accepted based on the study findings, which show that exposed dentine tubules treated with EB@TiO2 had excellent occlusion and acid resistance. Dentine tubule blockage was considerably different (p 0.001) in the samples treated with EB@TiO2, eggshell powder, and GIC Mousse. The observed discrepancies might be attributed to the particle sizes of EB@TiO2 (13 nm) and Eggshell powder (25 m), given the statistical difference between the eggshell powder and EB@TiO2 (p 0.008). Nano-sized calcium carbonate-containing materials, according to Nakashima et al. [9], might possibly remineralize injured teeth due to their specific features that allow adhesion to the oral surface. Calcium ions are discharged into the oral fluid as a result of this connection, obstructing dentinal tubules. Furthermore, GIC Mousse occluding capacities were shown to be substantially lower than eggshell powder (p 0.003) and EB@TiO2 (p 0.001), respectively. The samples treated with EB@TiO2 had the largest occlusion per area (83.2 12.4), whereas the group treated with GIC Mousse toothpaste had the lowest (25 8.04). These variations might be due to the toothpaste's composition and design. Nonetheless, the GIC Mousse-treated group's mean number of occluded dentinal tubules indicated that the toothpaste might still occlude dentinal tubules. The observed blockage of certain tubules in the samples treated with GIC Mousse may be due to the silica component in the toothpaste, as reported by Pashley et al. [20]. This backs up Wang et al. [21]'s findings that toothpastes containing abrasive ingredients such calcium carbonate and silica can produce a new smear layer on the dentine's surface, thus occluding the dentinal. The acid resistance of the treated samples was also assessed after they were treated with 2wt. percent citric acid.

The deposition of TiO2 on the calcite (eggshell) surface may have impacted the acid resistance of EB@TiO2 (Figure 1). This supports the findings of Tao et al. [14], who found that titanium dioxide can increase the acid resistance of calcium carbonates. On the other hand, Arnold et al. [10] noted that while some toothpastes, including such GIC Mousse, may enhance dentinal occlusion, such occlusion is of Nano/micro fluorhyhydroxy apatite crystals [22], nano hydroxy apatite/mesoporous [11] silica composite, and zinc oxide hydroxyapatite paste [23] have shown promising results in the treatment of DH, according to the literature reviewed for this study. Because the majority of the material is created from discarded eggshell, EB@TiO2 bio composite seemed to have the benefit of being easily available, but unlike aforementioned substances. Employing eggshell waste products to cure DH will improve the economic advantages connected with using natural waste products, which is prominent on the global agenda for a cleaner environment, as suggested by Onwubu et al. [24]. Yazcolu & Ulukap [25] endorse this position, stating that low-cost, accessible, practicable, and sustainable goods are needed to repair and enhance the quality of life for patients who haven't had to experience toothache due to sensitivity. This indicates that EB@TiO2 might be employed as an oral healthcare product in the treatment of DH. In addition, the cytotoxicity result (Figure 2) indicates that EB@TiO2 had no effect on the NIH 3T3 cell line. However, more in vivo study is required to thoroughly define the cytotoxicity as well as the occluding capability of the EB@TiO2 bio material. This is an area of study that needs to be explored. In addition, despite EB@TiO2's exceptional occluding properties, certain limitations were discovered in this work. The pace and number of days in an oral state necessary to totally occlude the tubules cannot be predicted using the agitation process. As a result, future research will examine the composite's occluding properties utilizing the brushing method recorded in the presence or absence of saliva. This research will aid in the development of the EB@TiO2 composite as a viable DH management material.

## Conclusions:

Data shows that the modified EB@TiO2 composite may successfully occlude dentine tubules, according to this investigation. The occlusion was also shown to have depth and be particularly effective in an acidic environment.
